# Nucleotide-binding oligomerization domain containing-like receptor family, caspase recruitment domain (CARD) containing 4 (NLRC4) regulates intrapulmonary replication of aerosolized *Legionella pneumophila*

**DOI:** 10.1186/1471-2334-13-371

**Published:** 2013-08-10

**Authors:** William R Berrington, Kelly D Smith, Shawn J Skerrett, Thomas R Hawn

**Affiliations:** 1 Department of Medicine, University of Washington School of Medicine, 1959 NE Pacific Street, Box 356423, Seattle, Washington, 98195-6523, USA

**Keywords:** *Legionella pneumophila*, Pneumonia, Alveolar macrophage, NLRC4, TLR5

## Abstract

**Background:**

*Legionella pneumophila (*Lp) flagellin activates signaling pathways in murine macrophages that control Lp replication. Nucleotide-binding oligomerization domain (NOD) containing-like receptor (NLR) family, caspase recruitment domain (CARD) containing 4 (NLRC4) and Toll-like Receptor (TLR5) both recognize Lp flagellin in vitro, but whether these two receptors play redundant or separate functional roles in vivo is unknown.

**Methods:**

The immune response of *Nlrc4−/−*, *Nlrc4−/−/Tlr5−/−*, and wild type C57Bl/6 mice was analyzed after in vivo infection with aerosolized Lp.

**Results:**

Lp clearance from the lungs was delayed in *Nlrc4−/−* mice over seven days in comparison to wild type controls. *Nlrc4−/−/Tlr5−/−* mice had no additional defect. In contrast to TLR5, NLRC4 did not regulate recruitment of neutrophils to the lung. Although there were no differences among the mouse strains in the lung transcriptome at 4 hours, *Nlrc4−/−* and *Nlrc4−/−Tlr5−/−* mice had increased lung inflammation at 72 hours in comparison to WT. *Nlrc4−/−/Tlr5−/−* mice also had altered cytokine production at both 4 and 24 hours post infection when compared to wild-type (WT) and *Nlrc4−/−* mice. Lp replication in murine alveolar macrophages was NLRC4-dependent and TLR5-independent.

**Conclusion:**

These studies reveal that NLRC4 and TLR5 mediate different roles in the inflammatory response to Lp flagellin in an aerosolized infection model and NLRC4 regulates replication in both lungs and alveolar macrophages.

## Background

*Legionella pneumophila* (Lp), an intracellular gram-negative rod, is a frequent cause of severe pneumonia [1]. Lp replicates and survives in the environment by infecting and persisting in waterborne amoeba and human infection occurs following exposure to aerosols of contaminated water [2]. Despite the discovery of Lp over three decades ago, we are just beginning to understand the role that innate recognition of molecules such as flagellin plays in host defense to the pathogen.

Lp is recognized by several classes of innate immune receptors that regulate different steps in the immune response. Extracellular and endosomal receptors (Toll-like receptor (TLR)-4, TLR2, TLR9 and TLR5) and cytoplasmic receptors, NLRC4, Neuronal apoptosis inhibitory protein-5 (NAIP5), Retinoic acid inducible gene – I, Melanoma differentiation associated protein-5, NLR family, pyrin domain containing-7 (NLRP7), NOD1, and NOD2) detect Lp in the mouse and regulate the immune response to the pathogen [3-13]. Two receptor complexes, TLR5 and NLRC4/NAIP, detect Lp flagellin (FlaA) [4,14-16]. Through a series of in vitro studies of Lp replication in murine bone marrow derived macrophages (BMDM), NAIP5 was identified as a key regulator of bacterial growth [17,18]. Later studies showed that NAIP5, NLRC4, and apoptosis-associated speck protein containing CARD (ASC) oligomerize into an inflammasome complex after recognition of cytoplasmic flagellin leading to enhanced caspase-1 dependent IL-1β secretion and pyroptotic cell death [10,11,14,16]. In addition in the mouse Lp flagellin is detected by multiple NAIP orthologs (NAIP5 and NAIP6), causing oligomerization of the NLRC4 inflammasome and proteolytic activation of caspase-1 [19,20]. While replication of Lp in cultured murine BMDM is controlled through activation of the NLRC4/NAIP inflammasome, the role of the inflammasome in mediating resistance to Lp in alveolar macrophages and in the lungs of whole animals is only partially understood. Respiratory challenge studies with wild type and flagellin-deficient (FlaA-) Lp indicated that flagellin recognition is important for control of Lp replication in the lungs [4,15,21,22]. The A/J mouse, which has impaired signaling through the NLRC4/NAIP5 inflammasome, permits intrapulmonary replication of Lp, in contrast to mice with intact NLRC4 inflammasome activation [23]. In addition, pulmonary clearance of Lp is impaired in *Nlrc4−/−* mice, a finding that is reproduced by flagellin deficient organisms [21,22]. Therefore we know that the NLRC4/NAIP5 inflammasome helps to clear Lp from the lung, but the mechanism of clearance is largely unknown, and whether other flagellin detectors such as TLR5 play a redundant role also is unknown.

FlaA expression in Lp is a major virulence factor and important in the early host response to the organism [15,24]. Lp tightly controls the expression of flagellin to enhance its infectivity and suppression of flagellin expression is important in intracellular replication in the macrophage [25,26]. Most experiments identifying flagellin as an important determinant in macrophages, however, have been performed in bone marrow derived macrophages differentiated ex vivo, and the role that tissue macrophages such as the alveolar macrophage play in control of Lp replication in vivo remains largely unknown.

In this study we examine the in vivo immune response to aerosolized Lp infection in *Nlrc4−/−* and *Nlrc4−/−/Tlr5−/−* mice to determine whether impaired signaling through two separate flagellin detection pathways influences host immune responses and Lp survival in vivo. In addition we identify that alveolar macrophages restrict Lp growth in an NLRC4-dependent, but not TLR5-dependent manner.

## Methods

### Bacteria

*L. pneumophila* Corby (serogroup 1) and *L. pneumophila* Corby flagellin deficient (ΔflaA) were gifts from K Heuner [26]. *L. pneumophila* Philadelphia-1 was obtained from the American Type Culture Collection (ATCC 33152).

### Animal model

*Tlr5−/−* and *Nlrc4−/−* C57Bl/6 mice strains were obtained from Drs. Shizuo Akira and Vishva Dixit, respectively [27,28], and were backcrossed at least six generations onto C57BL/6. Wild type C57BL/6 mice were purchased from the Jackson Laboratory (Bar Harbor, ME). Mice were housed in laminar flow cages with ad lib access to sterile food and water. Male and female mice were used in approximately equal numbers, and were 8–10 weeks of age at the time of experimentation. The University of Washington Institutional Animal Care and Use Committee approved all experiments.

### Airborne infection model

Mice were exposed to aerosolized bacteria in a whole animal exposure chamber as described previously [4,29]. Lp was cultured on buffered yeast charcoal extract (BYCE) agar plates for 4 days at 35°C. Colonies were harvested by rinsing plates with PBS. The pooled suspension was pelleted by centrifugation and resuspended in PBS to approximately 3×10^10^ cells/ml, as estimated by optical density. The slurry was transferred to twin jet nebulizers from Salter Laboratories (Arvin, CA USA) and mice were exposed to aerosolized bacteria for 30 minutes. To determine actual bacterial deposition in the lungs in each experiment, four sentinel animals were euthanized with intraperitonal pentobarbital and exsanguinated immediately after aerosol exposure. Left lungs were homogenized in PBS and serial dilutions in Mueller Hinton broth were plated onto BYCE agar. At subsequent time points, left lungs were similarly homogenized and cultured. Unused lung homogenates were mixed in lysis buffer containing protease inhibitors (Roche, NJ) on ice for 30 minutes, centrifuged at 2,500 rpm, and stored at −80°C for further use. Right lungs were used for histology and alveolar cell recruitment, as described [4,30]. Following cannulation of the trachea, right lungs were lavaged four times with 0.5 ml volumes of 0.9% sodium chloride containing 0.6 mM EDTA. Cell counts in bronchoalveolar lavage samples were determined with a hemocytometer, and differentials were determined by examining cytocentrifuge specimens stained with a modified Wright-Giemsa technique (Diff Quick, Dade Behring, Dudingen, Switzerland). After lavage, right lungs were fixed by instillation of 4% paraformaldehyde at 20 cm of pressure. Sections of paraffin-embedded tissue were stained with hematoxylin and eosin. 20 separate high power fields of lung sections were examined by a pathologist and scored to determine the percentage of airspaces with inflammation that involved the alveolar airspaces.

### Protein detection

Lung homogenate cytokine levels were determined using a multiplex fluorescent bead array system (Luminex 100). Briefly dilutions of homogenates were captured onto antibody-coated fluorochrome embedded microspheres and read using a flow based sorting and detection platform. Cytokine levels were measured using a sandwich ELISA technique (R&D systems) as previously described [4,30].

### Alveolar macrophage isolation and infection

Resident alveolar macrophages (AMs), were harvested from uninfected mice as previously described [4]. Cells pooled from 8–10 mice were suspended in RPMI 1640 supplemented with 10% heat-inactivated FCS, 100 U/ml penicillin, 100 ug/ml streptomycin, and 2 mM L-glutamine, counted in a hemocytometer, and the viability was determined by the exclusion of trypan blue. The cells were cultured in poly-L-lysine-treated 96 well plates at a density of 1 × 10^6^ viable AM/ml (0.85 ml/well). After a 2 h incubation at 37°C in humid air with 5% CO_2_, the adherent monolayers were washed five times with pre-warmed Hank’s balanced salt solution (HBSS) and re-fed with antibiotic-free medium. Lp was added at an MOI of 1 and the plates were centrifuged at 400 × g for 7 minutes, then incubated for 1 hour. The monolayers were washed 4 times with warm HBSS and re-fed with antibiotic-free medium. Immediately and after 24, 48, and 72 h incubation at 37°C in humid air with 5% CO_2_ triplicate monolayers were lysed by the addition of saponin to a concentration of 0.1%. The lysate was serially diluted in Mueller Hinton broth and quantitatively cultured on buffered charcoal yeast extract (BCYE) agar. Colonies were counted after 3 days of incubation at 37°C in 5% CO_2_.

### Lung RNA isolation

Mice exposed to aerosolized Lp 4 h previously and uninfected controls were euthanized with pentobarbital and exsanguinated by cardiac puncture. The pulmonary vascular bed was perfused with 10 ml cold PBS and both lungs were harvested into RNALater (Qiagen, Hamburg, Germany). Total lung RNA was isolated with an RNAeasy column (Qiagen, Hamburg, Germany). Reverse transcriptase was performed with Superscript III reverse transcriptase (Invitrogen, Carlsbad, CA) according to the manufacturer’s protocol. Total lung cDNA was applied to murine exon arrays (Affymetrix 430 2.0) (Affymetrix, Santa Clara, CA) containing 45,100 probes for 22,006 genes. Each probe was analyzed separately. Induced genes in lung mRNA were determined by comparing the expression values of the probes (which were analyzed separately) in infected and uninfected mice. Genes induced in infected wild type C57Bl/6 mice (n = 2) were compared to genes from infected in *Nlrc4−/−* mice (N = 2 each) as well as uninfected C57Bl/6 (n = 2) and *Nlrc4−/−* (n = 2) mice. Raw intensity values were background corrected, log2 transformed and quantile normalized and adjusted by robust multichip average (RMA) prior to analysis [31].

### Statistics

Data are expressed as mean ± standard error of mean (SEM). For comparisons of continuous variables among multiple groups, significance was determined using one-way ANOVA with bonferroni post-hoc test analysis. Comparisons between two groups was analyzed with two-tailed T tests. For mRNA expression arrays, a generalized linear model for microarray by limma (Bioconductor [32]) was used. Unadjusted significance was determined for expression arrays by an empirical Bayes moderated t-statistic as described [33] using the equations: (1) (WTstimulated-WTunstimulated)-(*Nlrc4−/−*stimulated - *Nlrc4−/−*unstimulated), (2) (WTstimulated-WTunstimulated), and (3) (*Nlrc4−/−*stimulated - *Nlrc4−/−*unstimulated). For adjusted p values, a false discovery rate of 5% was used for the analysis in the manner described by Benjamini and Hochberg [34]. The cutoff of significance for genes included in Additional file 1: Table S1 and Additional file 2: Table S2 was an adjusted P value of 0.01, no cutoff for log_2_ fold change was used. Gene set enrichment was done using publicly available software (Broad institute [35,36]), using a false discovery rate of 25%.

## Results

### NLRC4 regulates pulmonary clearance of Lp, but not cellular recruitment to the lungs

**Figure 1:**
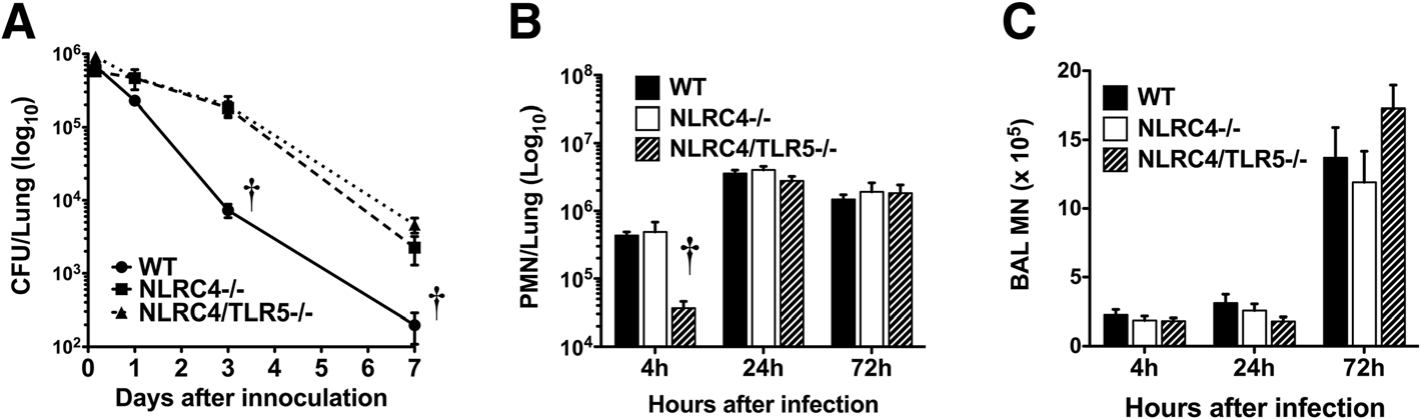
**NLRC4 regulates Lp replication, but not inflammatory cell recruitment to the lung after aerosolized infection.** WT (circles), *Nlrc4−/−* (squares), and *Nlrc4−/−/Tlr5−/−* mice (triangles) were exposed to aerosolized Lp Philadelphia-1 by aerosol infection (n = 8-12 for each time point). **(A)** Lp CFU measured at 4, 24 (1 day), 72 (3 days), and 168 (7 days) hours following infection **(B)** Lung neutrophil and **(C)** monocyte counts obtained by bronchoalveolar lavage of the right lung. Combined results from three separate experiments are depicted. Data are mean ± SEM † = p < 0.01 and * = p < 0.05 as determined by one-way ANOVA.

We infected WT, *Nlrc4−/−,* and *Tlr5−/−Nlrc4−/−* mice with aerosolized Lp to determine which immune responses are regulated by NLRC4 and TLR5 during pneumonic infection. *Nlrc4−/−* mice exhibited markedly delayed clearance of Lp from the lungs compared to WT mice; a 25-fold difference in lung CFUs was evident at the 72 hour time point and a 10-fold difference persisted to 10 days after infection (Figure 1A and data not shown (for day 10)). *Nlrc4−/−/Tlr5−/−* mice had no additional impairment in Lp clearance compared to the *Nlrc4−/−* animals (Figure 1A). Interestingly, Lp-infected *Nlrc4−/−* animals showed no difference in lung recruitment of neutrophils at 4, 24, or 72 hours in comparison to WT mice. In contrast, TLR5 and NLRC4 double knockout showed impaired neutrophil recruitment at 4 hours, similar to previously reported findings in *Tlr5−/−* animals (Figure 1B) [4]. These data demonstrate that NLRC4 is required for optimal clearance of Lp after airborne infection, but does not influence inflammatory cell recruitment to the lungs.

### NLRC4 and TLR5 regulate late cytokine production in Lp infected mice

**Figure 2:**
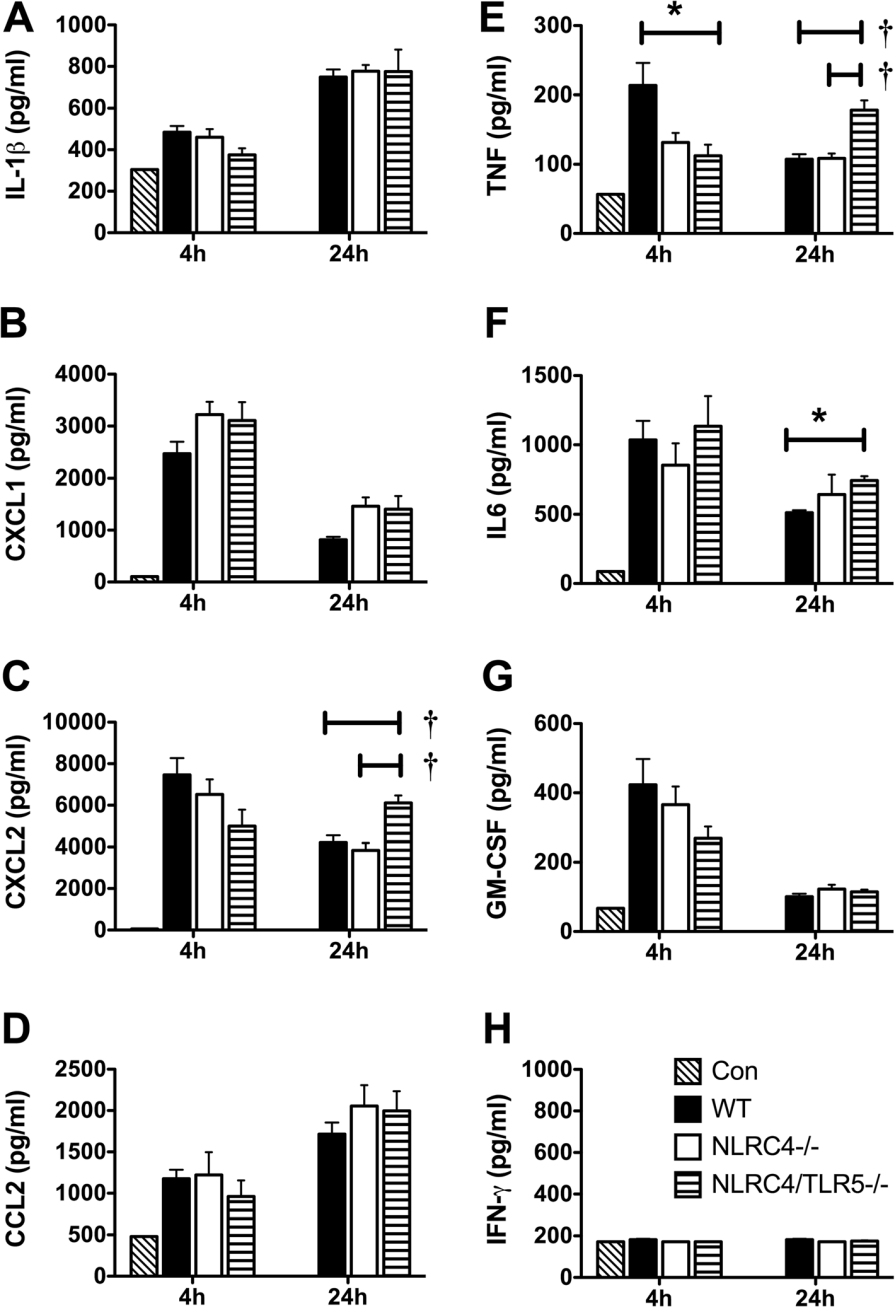
**Lung cytokine levels in mice infected with aerosolized*L. pneumophila*.** Cytokine levels in lung homogenates of mice infected with aerosol infection by Lp Phil-1. IL-1β **(A)**, CXCL1 **(B)**, CXCL2 **(C)**, CCL2 **(D)**, TNF **(E)**, IL-6 **(F)**, GM-CSF **(G)**, and IFN-γ **(H)** were measured by fluorescent bead immunoassay at 4 hours and 24 hours in untreated controls (diagonal hatched bars), WT (C57/Bl6) (black bars), *Nlrc4−/−* (white bars)*,* and *Nlrc4−/−/Tlr5−/−* (horizontal hatched bars) mice. Cytokine levels from uninfected controls are shown in (Con) lane at 4 h. Data is from n = 4-8 mice except uninfected control (n = 1). * = p < 0.05, † = p < 0.01 as determined by one-way ANOVA test.

Next we examined whether TLR5 and NLRC4 regulate cytokine production in the lungs of mice infected with Lp. At 4 h post-infection, the lungs of *Nlrc4−/−/Tlr5−/−* had decreased levels of tumor necrosis factor (TNF) compared to WT control lungs. These findings, however, were not consistent between two independent experiments (Figure 2E). Conversely at 24 hours post infection, TNF levels were consistently enhanced in *Nlrc4−/−/Tlr5−/−* mice when compared to *Nlrc4−/−* or WT mice (Figure 2E). In addition *Nlrc4−/−/Tlr5−/−* double knockout mice had significantly more proinflammatory chemokine (C-X-C motif) ligand 2 (CXCL2 or MIP-2) (Figure 2C) and interleukin 6 (IL-6) (Figure 2F) when compared to *Nlrc4−/−* (for CXCL2) or WT (for CXCL2 and IL-6) mice. No changes in lung levels of Interleukin 1-beta (IL-1β) (Figure 2A), chemokine (C-X-C motif) ligand 1 (CXCL-1 or mKC) (Figure 2B), chemokine (C-C motif) ligand 2 (CCL2 or MCP-1) (Figure 2D), GM-CSF (Figure 2G), and IFN- (Figure 2H) were detected at 4 h and 24 h when comparing WT, *Nlrc4−/−,* and *Nlrc4−/−/Tlr5−/−* mice. We also saw that during the 72 hour timepoint, there were increased cytokines (IL-1β, MIP2, CXCL-1, MCP-1, and IL-6) observed in Nlrc4-/-/Tlr5-/- mice (data not shown). Together, these data suggest that animals deficient in both NLRC4 and TLR5 pathways produce higher levels of lung pro-inflammatory cytokines than WT or NLRC4 single knockout animals.

### NLRC4 does not regulate early lung transcriptional responses

**Figure 3:**
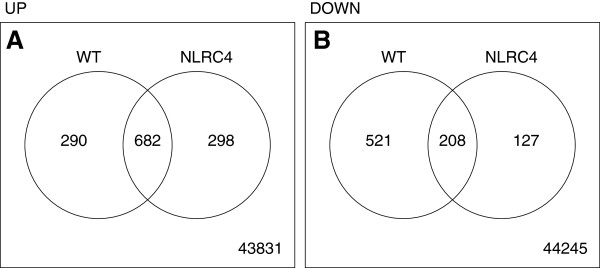
**Lung mRNA transcriptome after aerosolized Lp infection.** Whole lung RNA was isolated 4 hours following Lp infection of WT and *Nlrc4−/−* mice and analyzed for differential expression compared to uninfected controls by exon array analysis. 2 mice were analyzed per condition. Venn diagrams of genes increased **(A)** and decreased **(B)** compared to uninfected controls are depicted. Significantly different genes induced or suppressed by Lp infection in WT and *Nlrc4−/−* lungs are listed in Additional file 1: Table S1 and Additional file 2: Table S2 respectively. 3 genes were significantly increased or decreased in WT versus *Nlrc4−/−* lungs (Table 1). Significant difference in genes was determined by general linear model using a false discovery rate of 0.05 (see methods).

The NLRC4 inflammasome activates caspase-1 and also influences macrophage survival. However, little is known if genes downstream of NLRC4 control replication in the lungs during in vivo infection. To explore this, we isolated mRNA from the lungs of Lp-infected WT and *Nlrc4−/−* mice 4 hours after infection (n = 2) and measured expression levels with an Affymetrix 430 2.0 exon array. 1701 probes from 1275 genes were differentially regulated (972 probes were induced and 729 repressed) in the WT mice and 1315 probes from 978 genes (980 probes were induced and 335 repressed) in *Nlrc4−/−* animals compared to the uninfected controls (using a linear model fit from Bioconductor, p < 0.01 [33,37]) (Figure 3, Table 1, Additional file 1: Table S1, Additional file 2: Table S2, Additional file 3: Table S3, and Additional file 4: Table S4). When comparing infected animals, only 4 probes from 3 genes (D site albumin promoter binding protein, RIKEN cDNA G530011O06 gene, testis expressed gene 11) were different between WT and *Nlrc4−/−* mice (Table 1, using the formula [(WT_stim_ – WT_unstim_) - (*Nlrc4−/−*_stim_-*Nlrc4−/−*_unstim_)] with a generalized linear model with a false discovery rate of <5%) (Table 1). In addition gene set enrichment analysis (GSEA) was performed using a 25% false discovery rate cutoff and no gene sets were noted to be significantly different in the lungs of *Nlrc4−/−* mice compared to WT after infection with LP. Together, these data suggest that the whole lung transcriptional response to Lp is very similar between WT and *Nlrc4−/−* mice.

**Table 1:** **Genes significantly different between WT and*Nlrc4−/−*Lp-infected mice**

**Genes different between WT and*Nlrc4−/−*mice**	**Name**	**Fold change (95% CI), p**
Dbp	D site albumin promoter binding protein	0.05 (0.04-0.05), p = 6.62E-03
Tex11	Testis expressed gene 11	0.19 (0.16-0.21), p = 2.65E-03
Dbp	D site albumin promoter binding protein	0.04 (0.03-0.04), p = 5.62E-04
G530011O06Rik	RIKEN cDNA G530011O06 gene	46.41 (39.30-54.80), p = 2.51E-03
**Genes different between WT infected and uninfected mice**		
Slc26a4	Solute carrier family 26, member 4	52.83 (46.51-60.02), p = 1.37E-05
Cd14	CD14 antigen	15.25 (13.64-17.06), p = 1.58E-05
Cxcl1	Chemokine (C-X-C motif) ligand 1	166.48 (147.58-187.81), p = 1.73E-05
Ereg	Epiregulin	11.31 (10.11-12.64), p = 2.07E-05
Gm1960	Gene model 1960, (NCBI)	383.16 (331.67-442.63), p = 2.07E-05
Cxcl1	Chemokine (C-X-C motif) ligand 1	56.19 (49.78-63.43), p = 2.07E-05
Steap4	STEAP family member 4	14.71 (13.02-16.63), p = 2.07E-05
G530011O06Rik	RIKEN cDNA G530011O06 gene	72.30 (61.82-84.56), p = 2.48E-05
Il4i1	Interleukin 4 induced 1	41.87 (33.79-51.89), p = 3.09E-05
Cxcl2	Chemokine (C-X-C motif) ligand 2	399.39 (355.05-449.26), p = 3.32E-05
**Genes different between*Nlrc4−/−*infected and uninfected mice**		
Tbl3	Transducin (beta)-like 3	2.09 (1.85-2.37), p = 2.93E-03
Gja1	Gap junction membrane channel protein alpha 1	2.50 (2.22-2.81), p = 6.04E-04
Gja1	Gap junction membrane channel protein alpha 1	3.39 (3.04-3.78), p = 3.75E-04
Slc16a1	Solute carrier family 16 (monocarboxylic acid transporters), member 1	1.53 (1.29-1.80), p = 8.34E-03
Sfrs2	Splicing factor, arginine/serine-rich 2 (SC-35)	2.06 (1.79-2.37), p = 9.27E-04
Scd2	Stearoyl-Coenzyme A desaturase 2	0.55 (0.49-0.63), p = 3.51E-03
Scd2	Stearoyl-Coenzyme A desaturase 2	0.48 (0.43-0.54), p = 2.79E-03
Aldh18a1	Aldehyde dehydrogenase 18 family, member A1	1.86 (1.66-2.08), p = 6.23E-03
Junb	Jun-B oncogene	5.19 (4.66-5.79), p = 2.32E-04
Marcksl1	MARCKS-like 1	4.94 (4.44-5.50), p = 2.31E-04

Depicted above are the top ten most significant genes differentially expressed in 1) *Nlrc4−/−* vs *Tlr5−/−*, 2) WT infected and uninfected and 3) *Nlrc4−/−* infected and uninfected as determined by a general linear model using a false discovery rate of 0.05.

Complete gene lists are included in online supplemental material to highlight similarities between probes differentially regulated between WT and NLRC4−/− infected lungs.

### NLRC4 regulates lung inflammation

**Figure 4:**
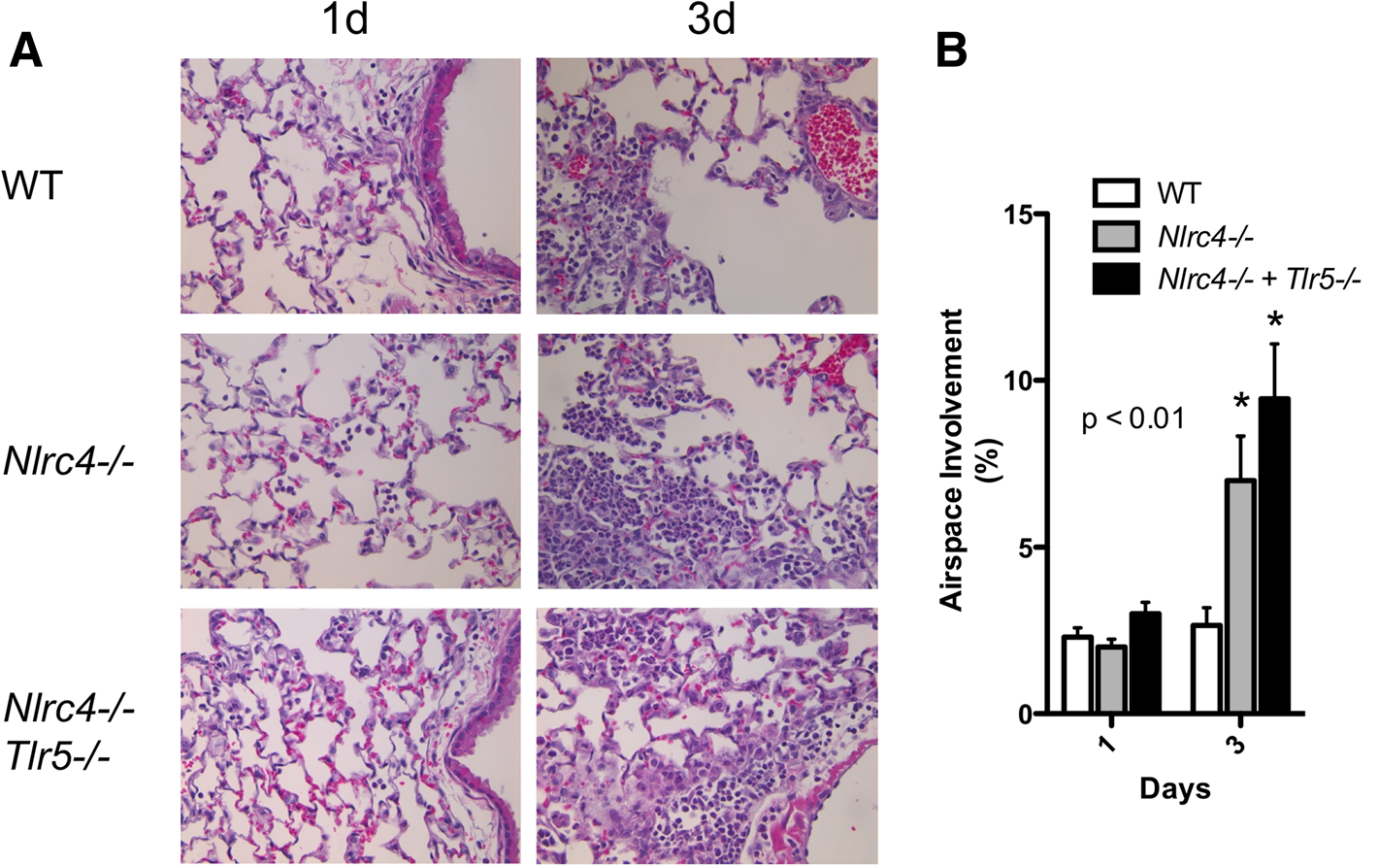
**Lung histology in*Nlrc4−/−*and*Nlrc4−/−/Tlr5−/−*mice after aerosolized Lp infection. (A)** Lungs were isolated from mice infected with aerosolized Lp and stained with Hematoxylin and Eosin. Graphic depiction of inflammation scored by pathologist is shown in **(B)** p < 0.01 by 2 way ANOVA for data analysis, scoring 10 separate fields from 2 different lungs from WT (open columns), *Nlrc4−/−* (grey), and *Nlrc4−/−/Tlr5−/−* mice.

We next examined lung histology in WT, *Nlrc4−/−,* and *Nlrc4−/−/Tlr5−/−* mice at 1 and 3 days after infection and determined the percentage of inflamed airspace (Figure 4A). No difference was seen between WT and *Nlrc4−/−,* and *Nlrc4−/−/Tlr5−/−* mice at 24 hours following aerosol inoculation (Figure 4A and B). At 3 days following inoculation, WT lungs showed significantly less inflammation then the *Nlrc4−/−* and *Nlrc4−/−/Tlr5−/−* animals (Figure 4B). Compared to WT mice, the *Nlrc4−/−* and *Nlrc4−/−/Tlr5−/−* mice had increased patchy infiltration of the alveolar space by neutrophils with some macrophages, as is characteristic for Lp pneumonia in humans. No significant difference was seen between *Nlrc4−/−* and *Nlrc4−/−/Tlr5−/−* mice. These data suggest that increased replication in *Nlrc4−/−* mice is associated with an increased inflammatory response in the lungs 3 days following Lp exposure.

### NLRC4, but not TLR5, controls intracellular replication in alveolar macrophages

**Figure 5:**
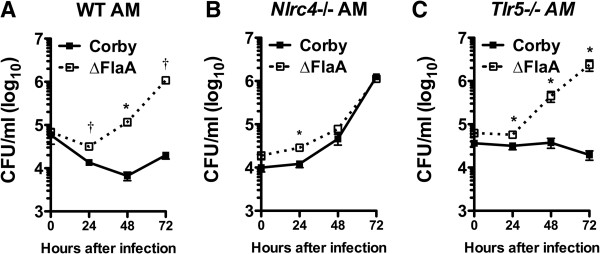
**NLRC4 deficiency impairs replication in murine alveolar macrophages.** Alveolar macrophages (AM) were isolated from lungs of WT **(A)**, *Nlrc4−/−***(B)**, and *Tlr5−/−***(C)** mice by bronchoalveolar lavage of uninfected mice. Cells were infected *ex vivo* with WT (Corby) Lp or isogenic FlaA- (Corby) Lp and CFUs were measured in lysed monolayers at 24, 48, and 72 hours. Data are mean ± SEM of triplicate or quadruplicate monolayers at each time point (except for duplicate measures at time 0 from TLR5−/− mice.). *p < 0.05, †p < 0.005 as determined by two-tailed Students’ t-test.

To examine possible mechanisms of NLRC4-dependent Lp replication in lungs, we next measured Lp replication in alveolar macrophages. We infected WT, *Nlrc4−/−* and *Tlr5−/−* alveolar macrophages with LpWT or LpFlaA- and examined replication over 72 hours. Murine alveolar macrophages restricted LpWT growth (Figure 5) in a NLRC4, but not TLR5, dependent manner (Figure 5 A-C). Alveolar macrophages harvested from WT and TLR5-deficient mice supported replication of LpFlaA- but not LpWT, whereas both Lp strains replicated alveolar macrophages from NLRC4-null mice (Figure 5A-C). These data indicate that NLRC4, but not TLR5, restricts the growth of Lp in alveolar macrophages, and suggest that flagellin induces NLRC4-mediated resistance to Lp in murine alveolar macrophages.

## Discussion

Our primary finding is that TLR5 and NLRC4 mediate different roles in the inflammatory response to Lp flagellin in an aerosolized infection model. In earlier studies, TLR5 detection of Lp flagellin was shown to regulate early recruitment of PMNs to the alveolar space that results in organizing pneumonia at later stages without a difference in bacterial replication [4]. In this study, we again observed that TLR5 regulates early recruitment of PMNs to the alveolar space but not bacterial replication in *Nlrc4−/−/Tlr5−/−* mice compared to *Nlrc4−/−* and WT mice. In contrast to TLR5, we show here that NLRC4 regulates bacterial replication in vitro in alveolar macrophages and in vivo by accelerating Lp clearance from the lungs.

NLRC4 control of in vivo Lp growth could be mediated via several mechanisms including pyroptosis or via IL-1β or IL-18-dependent mechanisms. Previous in vitro studies in murine BMDM demonstrated that control of Lp replication is NLRC4/NAIP5-dependent and likely mediated through regulation of pyroptosis [14]. Therefore increased growth of Lp in the lungs of *Nlrc4−/−* and *Nlrc4−/−/Tlr5−/−* mice may be due to enhanced replication or failure of alveolar macrophage to undergo pyroptosis in NLRC4-deficient lungs. Alternatively elevated CFUs in *Nlrc4−/−* and *Nlrc4−/−/Tlr5−/−* mice could also be explained by enhanced levels of IL-1β or IL-18 in WT animals. Although we found no difference in IL-1β production in lungs of Lp-infected mice WT mice compared to *Nlrc4−/−* and *Nlrc4−/−/Tlr5−/−*[38]. Transcriptional analysis of the array data reveal that IL-1β is transcriptionally increased at similar levels in both animals, our methods, which involve measurement of cytokines in lung homogenates, may not adequately distinguish cleaved bioactive IL-1β from pro-IL-1β *Nlrc4−/−* and WT animals infected by Lp, suggesting that early induction of pro-IL-1β is found in both WT and *Nlrc4−/−* animals. In support of a possible IL-18-dependent mechanism, a previous study demonstrated that IL-18 blockade with monoclonal antibodies contributes to control of Lp in the lungs when IL-12 is concurrently blocked, likely through an IFN-γ dependent mechanism [35]. However, others have found that *Il1*β*−/−/Il18−/−* mice are not more susceptible to Lp intraperitoneal infection when compared to WT mice [39]. Such a mechanism could contribute to NLRC4-dependent regulation of Lp growth during pulmonary infection.

In addition our study reveals elevated TNF and CXCL2 at 24 hours post-infection in *Nlrc4−/−/Tlr5−/−* mice compared to *Nlrc4−/−* and WT mice. The mechanisms underlying cytokine suppression seen in *Nlrc4−/−* animals compared to *Nlrc4−/−/Tlr5−/−* animals are currently only speculative. Perhaps signaling through TLR5 may evoke an anti-inflammatory response by causing refractory signaling in TLR signaling pathways at later timepoints (24 h). Another explanation may be that early TLR5-mediated neutrophil recruitment seen in WT mice may help to contain the inflammatory response either by eliciting an anti-inflammatory response (for example, by eferocytosis of apoptotic PMNs [40]) or by clearing a pro-inflammatory stimulus (presumably by phagoctosis).

We also observed that NLRC4-deficient animals have increased alveolar space inflammation at 72 h post-infection. One possible explanation for these findings is that failure to control Lp replication in alveolar macrophages of *Nlrc4−/−* animals contributes to delayed pulmonary clearance of Lp. The higher bacterial burden would therefore enhance later recruitment of lung inflammation by CFU-dependent increases in signaling through other pathways (such as Myd88-dependent pathways) known to be activated by Lp [5]. At 72 hours, the increase in inflammatory cells seen histologically in the airways of *Nlrc4−/−* mice is different from the bronchoalveolar lavage data that has no difference. The likely explanation for this difference is that the BAL fluid samples cells that are easily recovered from the conducting airways and the alveolar lumina, while the histology measures the aggregates of inflammatory cells that occupy the alveoli and may not be readily dislodged and sampled during lavage. Our findings are consistent with a delayed Lp clearance model with increased bacterial loads even at early time points in *Nlrc4−/−* mice compared to WT.

In our study we show that growth of Lp in alveolar macrophages is NLRC4-dependent and TLR5-independent, and provides a plausible cellular mechanism for the increased in vivo susceptibility of *Nlrc4−/−* mice to Lp. Murine alveolar macrophages differ from other primary and BMDMs in their ability to sense flagellin in a TLR5-dependent manner [4]. Despite these unique features of TLR5 in alveolar macrophages, our data show that control of Lp replication is not influenced by the presence of TLR5. Furthermore, our observations suggest that flagellin induces NLRC4-mediated resistance to Lp in murine alveolar macrophages, as has been described for BMDMs [10,11,15,16,19,20]. The implications of these data for human biology are not clear. Previous work has shown that human alveolar macrophages are more permissive to Lp replication than blood-derived monocytes [41]. Moreover, human alveolar macrophages differ from monocytes in the requirements for activation of the NLRC4 inflammasome. Alveolar macrophages lack constitutively activated caspase-1 and require a second stimulus for IL-1β secretion in response to TLR ligands, in contrast to monocytes [42-44]. Lp growth in human alveolar macrophages is at least partially NLRC4 and NAIP-dependent as determined by enhancement of Lp growth by siRNA knockdown of NLRC4 or NAIP in vitro [45]. Furthermore Lp has been shown to enhance the transcription of β-defensin-3 in a TLR5-dependent manner, which may influence Lp replication in humans differently from the mouse [46,47]. Whether TLR5 knockdown regulates replication of Lp in human AMs is unknown. The precise molecular mechanism of NLRC4 and NAIP control in the both murine and human alveolar macrophage is the focus of current research efforts.

Messenger RNA arrays performed on genes expressed in whole lung showed no difference between *Nlrc4−/−* and WT animals suggesting that NLRC4 inflammasome signaling at 4 hours is independent of transcription and may occur by post-translational mechanisms. After recognition of flagellin, the NLRC4/NAIP5 inflammasome oligomerizes and activates a proteolytic cascade beginning with activation of caspase-1. The lack of transcriptional regulation of NAIP5/NLRC4 inflammasome accompanied by the activation of proteolytic cascades by Lp has been observed previously at 4 hours in vitro in BMDMs isolated from C57Bl/6 mice compared to the A/J strain [48]. A separate study demonstrated that functionally deficient NAIP derived from A/J mice may regulate transcriptional signaling through IRF1 and IRF8 [49]. Due to experimental differences, it is difficult to compare our data with these previous findings (bone marrow derived macrophages infected ex vivo with Lp versus whole lung mRNA after in vivo aerosol infection). Our study is also potentially limited by power, and it is possible that significant differences in the transcriptome might be obscured by the small sample size. The absence of mRNA differences seen in vivo suggest that the observed phenotype is from post-translational, caspase-1 dependent events such as pyroptosis. Induction of NLRC4-mediated pyroptosis by Lp may enhance bacterial release by susceptible macrophages and permit phagocytosis by newly recruited neutrophils. This may promote bacterial clearance in the alveolar space, as seen in other models of intracellular infection [39]. Whether pyroptosis contributes to resistance to pneumonic legionellosis is currently unknown.

## Conclusions

These studies reveal that NLRC4 and TLR5 control different responses to Lp flagellin in an aerosolized murine infection model and NLRC4 regulates replication in both lungs and alveolar macrophages.

## Abbreviations

AM: Alveolar macrophage; ASC: Apoptosis-associated speck protein containing caspase recruitment domain; BCYE: Buffered charcoal yeast extract; BMDM: Bone marrow derived macrophages; CARD: Caspase recruitment domain; CFU: Colony forming unit; CI: Confidence interval; CXCL: Chemokine (C-X-C motif) ligand; CCL: Chemokine (C-C motif) ligand; FlaA-: Flagellin deficient; Lp: *Legionella pneumophila*; MOI: Multiplicity of infection; NAIP: Neuronal apoptosis inhibitory protein; NLR: Nucleotide-binding oligomerization domain-like receptor; NLRC4: Nucleotide-binding oligomerization domain containing-like receptor family, caspase recruitment domain (CARD) containing 4; NOD: Nucleotide-binding oligomerization domain; RIKEN: Rikagaku Kenkyusho; RMA: Robust multichip average; SEM: Standard error of mean; siRNA: Small inhibitory RNA; WT: Wild-type.

## Competing interests

The authors declare that they have no competing interests.

## Authors’ contributions

WRB conceived and designed the study, carried out the molecular array studies, performed the statistical analysis, and drafted the manuscript. SJS conceived and designed the study and performed the in vivo infection, studies, and edited the manuscript. KDS examined the lung histology and edited the manuscript. TRH conceived and designed the study and helped with drafting the manuscript and statistical analysis. All authors read and approved the final manuscript.

## Pre-publication history

The pre-publication history for this paper can be accessed here:

http://www.biomedcentral.com/1471-2334/13/371/prepub

## Supplementary Material

Additional file 1: Table S1 Genes differentially expressed in WT lungs after aerosol infection with Lp. Genes in this list had differentially expressed mRNA in WT Lp-infected lungs compared to uninfected controls. Log_2_ fold change was determined by the averaged difference between the Log_2_ mRNA values in lungs infected with Lp (WTstim) and uninfected lungs (WTunstim). Average expression in WTstim and WTunstim is the average of Log_2_ transformed mRNA expression values in two mice. Genes named “---” had no identifiers in probeset. Probe identifiers are from Affymetrix 2.0 array designations. Unadjusted p values were determined as described in methods. Adjusted p values were determined using a false discovery rate of 5% in the method of Benjamini and Hochberg (see methods). Click here for file

Additional file 2: Table S2 Genes differentially expressed in *Nlrc4−/−* lungs after aerosol infection with Lp. Genes in this list had differentially expressed mRNA in *Nlrc4−/−* Lp-infected lungs compared to uninfected *Nlrc4−/−* controls. Log_2_ fold change was determined by the averaged difference between the Log_2_ mRNA values in lungs infected with Lp (*Nlrc4−/−*_stim_) and uninfected lungs (*Nlrc4−/−*_unstim_). Average expression in *Nlrc4−/−*_stim_ and *Nlrc4−/−*_unstim_ is the average of Log_2_ transformed mRNA expression values in two mice. Genes named “---” had no naming identfication. Probe identifiers are from Affymetrix 2.0 array designations. Unadjusted p values were determined as described in methods. Adjusted p values were determined using a false discovery rate of 5% in the method of Benjamini and Hochberg (see methods). Click here for file

Additional file 3: Table S3 Genes signficantly decreased in WT Lp-infected lungs but not significant in *Nlrc4−/−* mice. Click here for file

Additional file 4: Table S4 Genes signficantly increased in WT Lp-infected lungs but not significant in *Nlrc4−/−* mice. Click here for file
